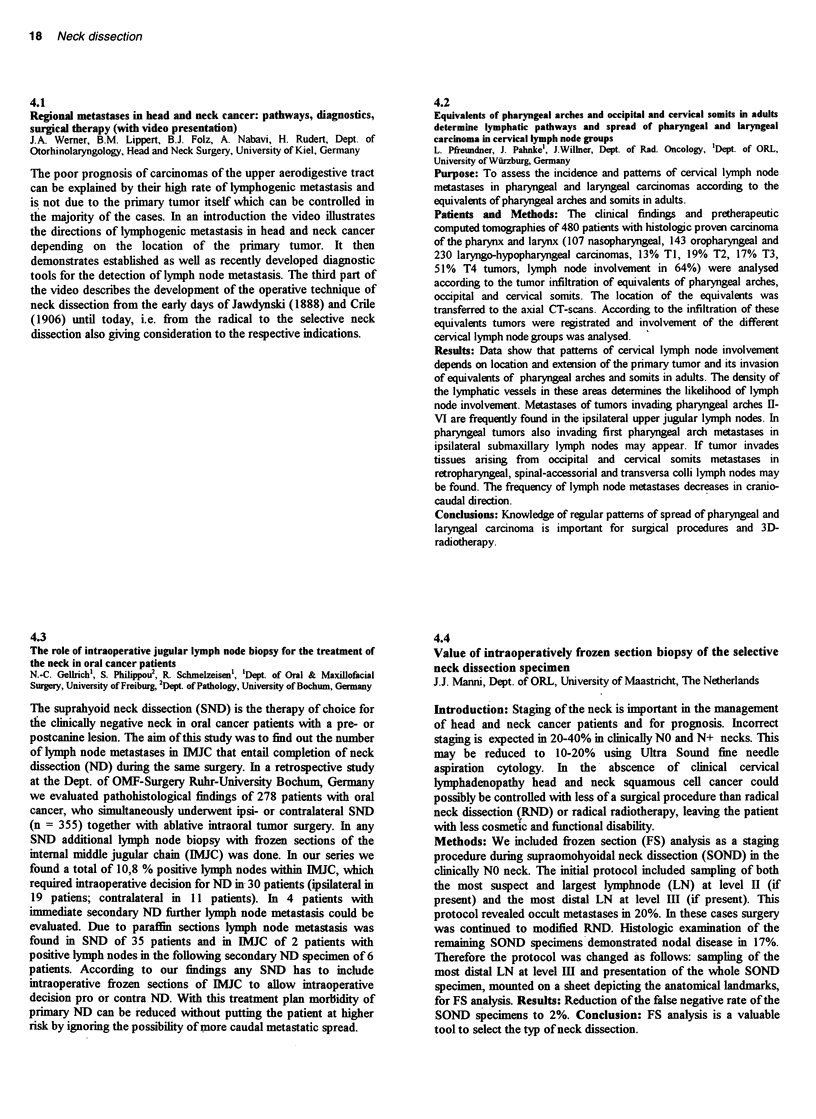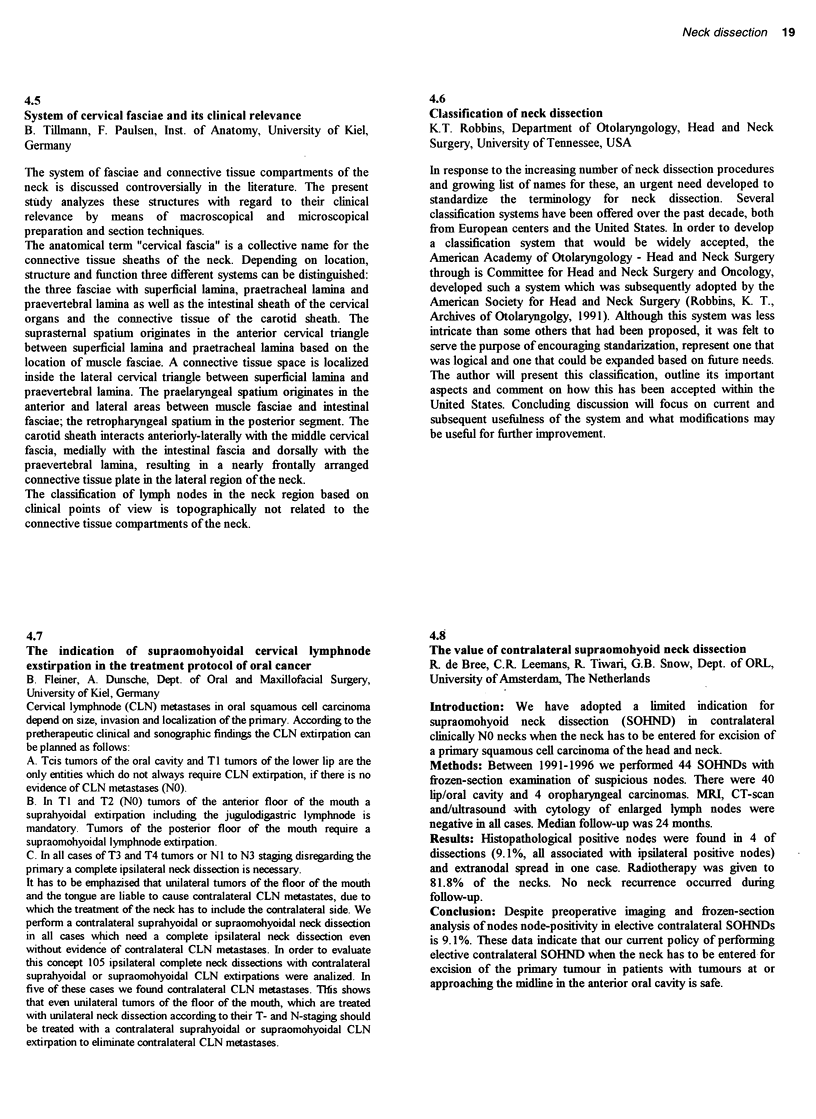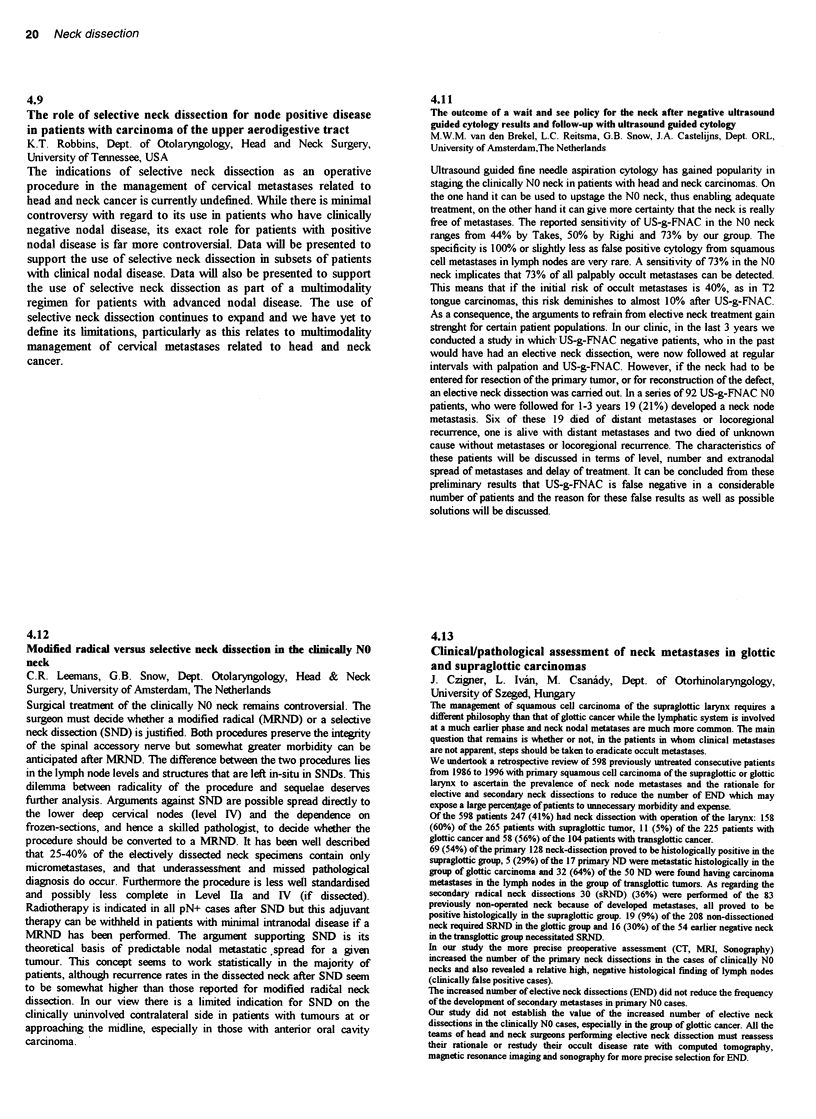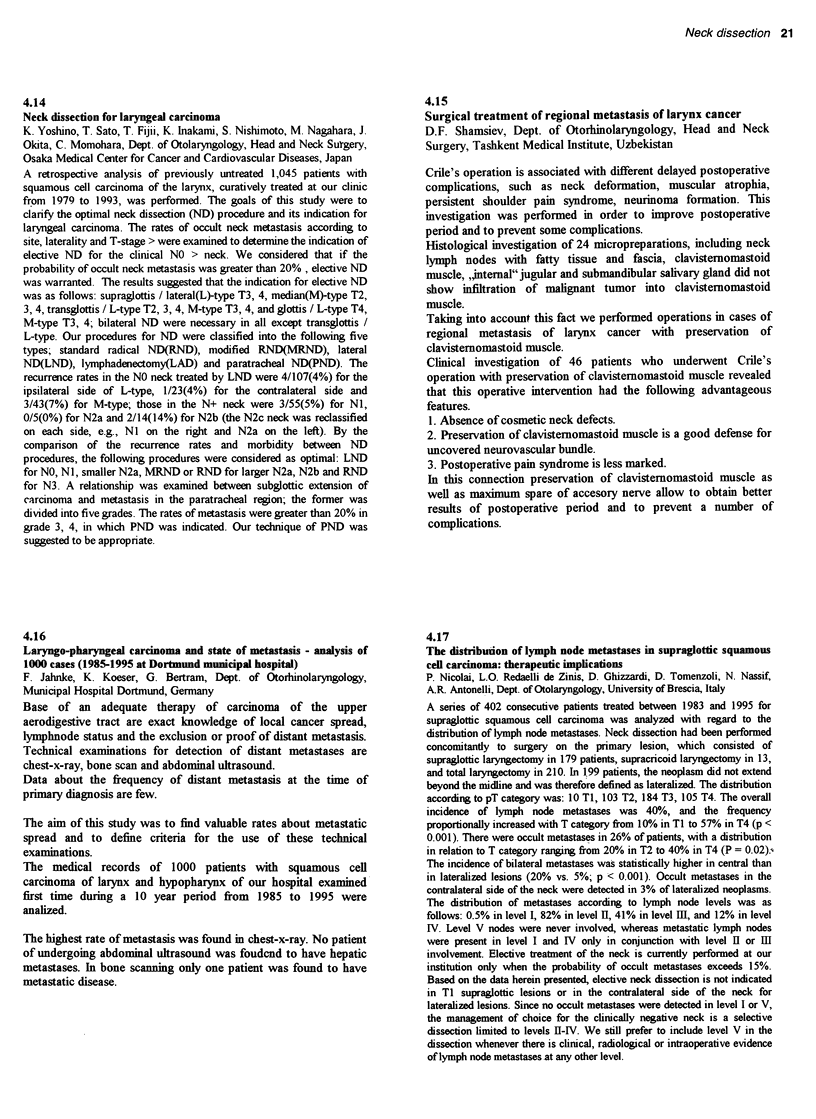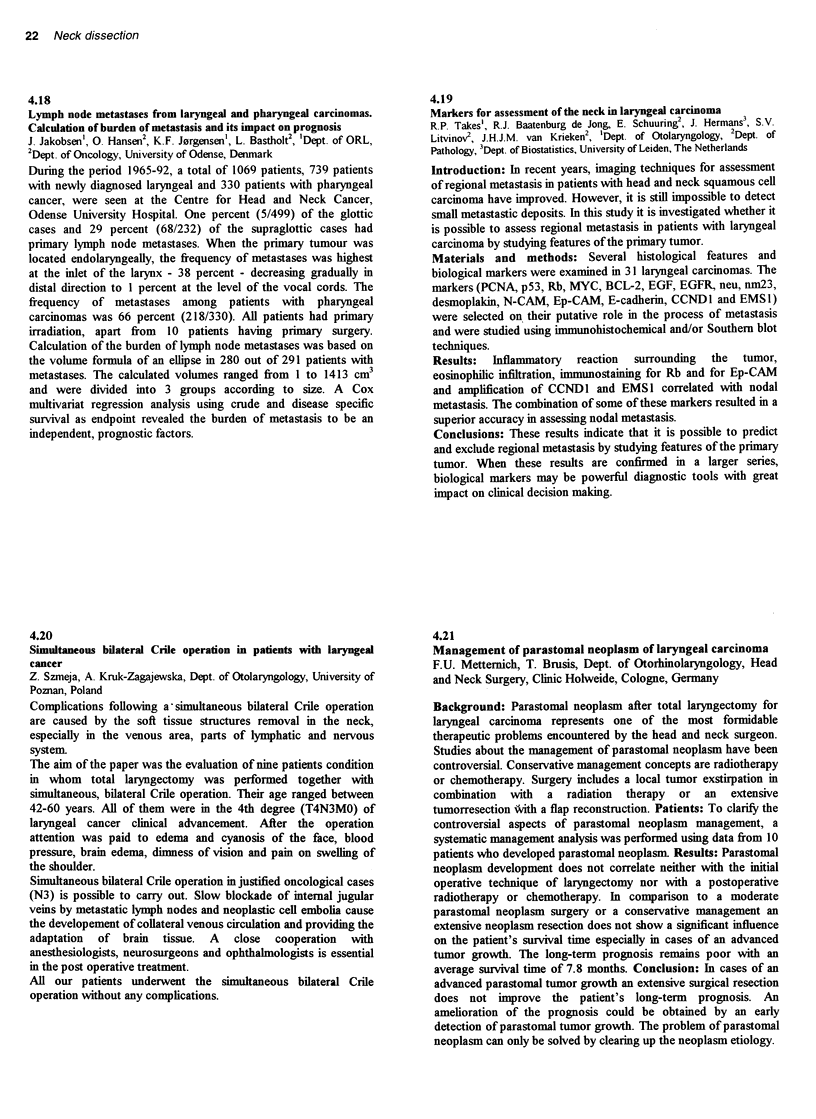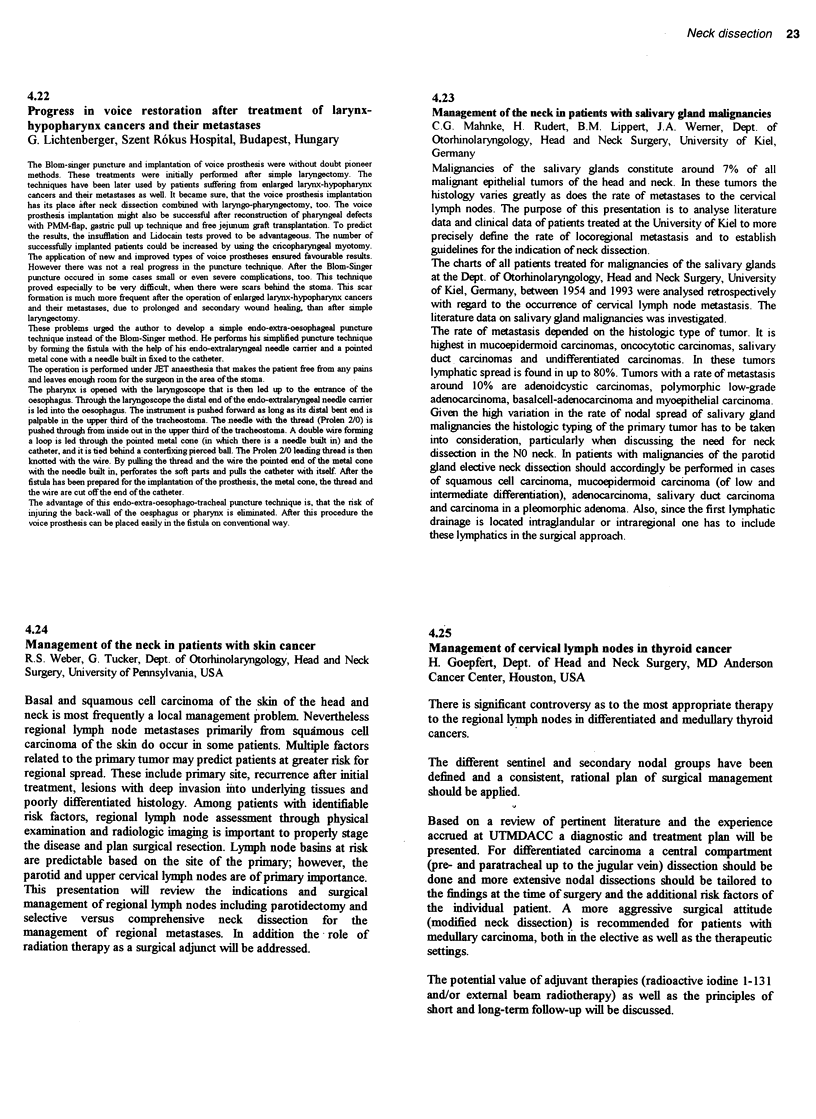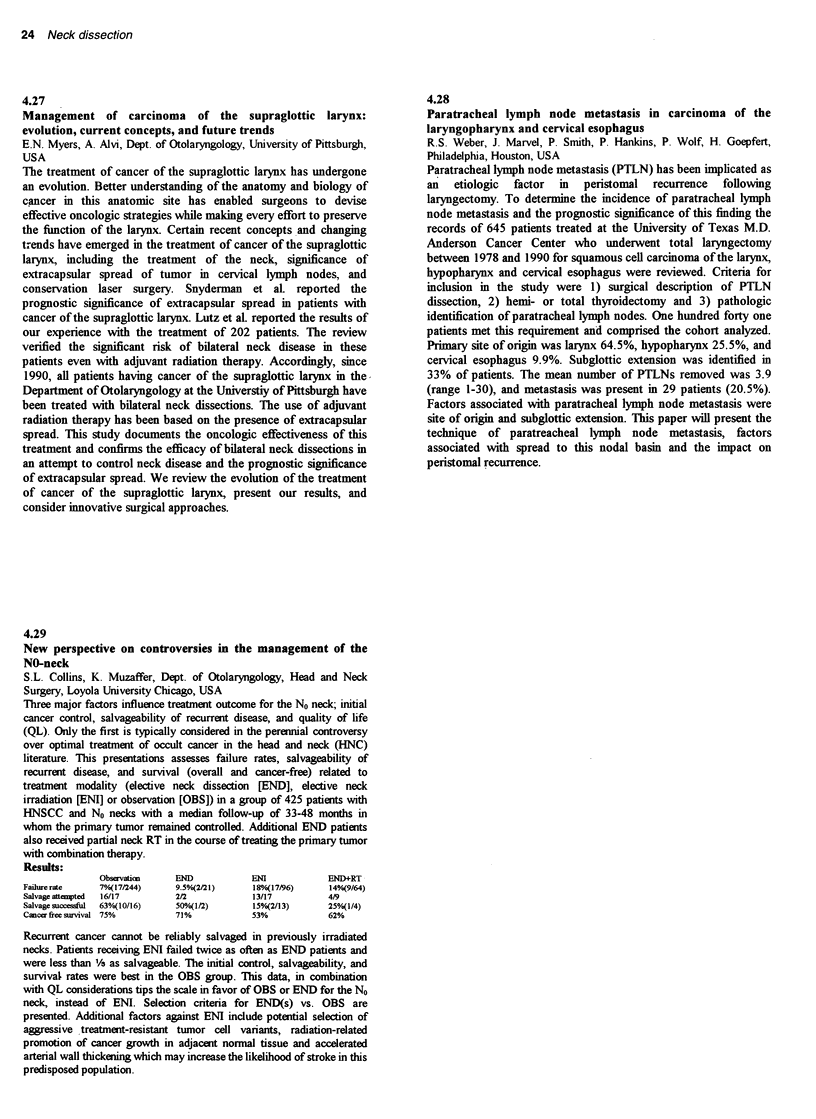# Neck Dissection

**Published:** 1998

**Authors:** 


### 4.1
**Regional metastases in head and neck cancer: pathways, diagnostics, surgical therapy (with video presentation)**

J.A. Werner, B.M. Lippert, B.J. Folz, A. Nabavi, H. Rudert, Dept. of Otorhinolaryngology, Head and Neck Surgery, University of Kiel, Germany

The poor prognosis of carcinomas of the upper aerodigestive tract can be explained by their high rate of lymphogenic metastasis and is not due to the primary tumor itself which can be controlled in the majority of the cases. In an introduction the video illustrates the directions of lymphogenic metastasis in head and neck cancer depending on the location of the primary tumor. It then demonstrates established as well as recently developed diagnostic tools for the detection of lymph node metastasis. The third part of the video describes the development of the operative technique of neck dissection from the early days of Jawdynski (1888) and Crile (1906) until today, i.e. from the radical to the selective neck dissection also giving consideration to the respective indications.

### 4.2
**Equivalents of pharyngeal arches and occipital and cervical somits in adults determine lymphatic pathways and spread of pharyngeal and laryngeal carcinoma in cervical lymph node groups**

L. Pfreundner, J. Pahnke[1], J.Willner, Dept. of Rad. Oncology, [1]Dept. of ORL, University of Würzburg, Germany

**Purpose:** To assess the incidence and patterns of cervical lymph node metastases in pharyngeal and laryngeal carcinomas according to the equivalents of pharyngeal arches and somits in adults.

**Patients and Methods:** The clinical findings and pretherapeutic computed tomographies of 480 patients with histologic proven carcinoma of the pharynx and larynx (107 nasopharyngeal, 143 oropharyngeal and 230 laryngo-hypopharyngeal carcinomas, 13% T1, 19% T2, 17% T3, 51% T4 tumors, lymph node involvement in 64%) were analysed according to the tumor infiltration of equivalents of pharyngeal arches, occipital and cervical somits. The location of the equivalents was transferred to the axial CT-scans. According to the infiltration of these equivalents tumors were registrated and involvement of the different cervical lymph node groups was analysed.

**Results:** Data show that patterns of cervical lymph node involvement depends on location and extension of the primary tumor and its invasion of equivalents of pharyngeal arches and somits in adults. The density of the lymphatic vessels in these areas determines the likelihood of lymph node involvement. Metastases of tumors invading pharyngeal arches II-VI are frequently found in the ipsilateral upper jugular lymph nodes. In pharyngeal tumors also invading first pharyngeal arch metastases in ipsilateral submaxillary lymph nodes may appear. If tumor invades tissues arising from occipital and cervical somits metastases in retropharyngeal, spinal-accessorial and transversa colli lymph nodes may be found. The frequency of lymph node metastases decreases in cranio-caudal direction.

**Conclusions:** Knowledge of regular patterns of spread of pharyngeal and laryngeal carcinoma is important for surgical procedures and 3D-radiotherapy.

### 4.3
**The role of intraoperative jugular lymph node biopsy for the treatment of the neck in oral cancer patients**

N.-C. Gellrich[1], S. Philippou[2], R. Schmelzeisen[1], [1]Dept. of Oral & Maxillofacial Surgery, University of Freiburg, [2]Dept. of Pathology, University of Bochum, Germany

The suprahyoid neck dissection (SND) is the therapy of choice for the clinically negative neck in oral cancer patients with a pre- or postcanine lesion. The aim of this study was to find out the number of lymph node metastases in IMJC that entail completion of neck dissection (ND) during the same surgery. In a retrospective study at the Dept. of OMF-Surgery Ruhr-University Bochum, Germany we evaluated pathohistological findings of 278 patients with oral cancer, who simultaneously underwent ipsi- or contralateral SND (n = 355) together with ablative intraoral tumor surgery. In any SND additional lymph node biopsy with frozen sections of the internal middle jugular chain (IMJC) was done. In our series we found a total of 10,8 % positive lymph nodes within IMJC, which required intraoperative decision for ND in 30 patients (ipsilateral in 19 patiens; contralateral in 11 patients). In 4 patients with immediate secondary ND further lymph node metastasis could be evaluated. Due to paraffin sections lymph node metastasis was found in SND of 35 patients and in IMJC of 2 patients with positive lymph nodes in the following secondary ND specimen of 6 patients. According to our findings any SND has to include intraoperative frozen sections of IMJC to allow intraoperative decision pro or contra ND. With this treatment plan morbidity of primary ND can be reduced without putting the patient at higher risk by ignoring the possibility of more caudal metastatic spread.

### 4.4
**Value of intraoperatively frozen section biopsy of the selective neck dissection specimen**

J.J. Manni, Dept. of ORL, University of Maastricht, The Netherlands

**Introduction:** Staging of the neck is important in the management of head and neck cancer patients and for prognosis. Incorrect staging is expected in 20-40% in clinically N0 and N+ necks. This may be reduced to 10-20% using Ultra Sound fine needle aspiration cytology. In the abscence of clinical cervical lymphadenopathy head and neck squamous cell cancer could possibly be controlled with less of a surgical procedure than radical neck dissection (RND) or radical radiotherapy, leaving the patient with less cosmetic and functional disability.

**Methods:** We included frozen section (FS) analysis as a staging procedure during supraomohyoidal neck dissection (SOND) in the clinically N0 neck. The initial protocol included sampling of both the most suspect and largest lymphnode (LN) at level II (if present) and the most distal LN at level III (if present). This protocol revealed occult metastases in 20%. In these cases surgery was continued to modified RND. Histologic examination of the remaining SOND specimens demonstrated nodal disease in 17%. Therefore the protocol was changed as follows: sampling of the most distal LN at level III and presentation of the whole SOND specimen, mounted on a sheet depicting the anatomical landmarks, for FS analysis. **Results:** Reduction of the false negative rate of the SOND specimens to 2%. **Conclusion:** FS analysis is a valuable tool to select the typ of neck dissection.

### 4.5
### System of cervical fasciae and its clinical relevance
B. Tillmann, F. Paulsen, Inst. of Anatomy, University of Kiel, Germany

The system of fasciae and connective tissue compartments of the neck is discussed controversially in the literature. The present study analyzes these structures with regard to their clinical relevance by means of macroscopical and microscopical preparation and section techniques.
The anatomical term "cervical fascia" is a collective name for the connective tissue sheaths of the neck. Depending on location, structure and function three different systems can be distinguished: the three fasciae with superficial lamina, praetracheal lamina and praevertebral lamina as well as the intestinal sheath of the cervical organs and the connective tissue of the carotid sheath. The suprasternal spatium originates in the anterior cervical triangle between superficial lamina and praetracheal lamina based on the location of muscle fasciae. A connective tissue space is localized inside the lateral cervical triangle between superficial lamina and praevertebral lamina. The praelaryngeal spatium originates in the anterior and lateral areas between muscle fasciae and intestinal fasciae; the retropharyngeal spatium in the posterior segment. The carotid sheath interacts anteriorly-laterally with the middle cervical fascia, medially with the intestinal fascia and dorsally with the praevertebral lamina, resulting in a nearly frontally arranged connective tissue plate in the lateral region of the neck.
The classification of lymph nodes in the neck region based on clinical points of view is topographically not related to the connective tissue compartments of the neck.

### 4.6
### Classification of neck dissection
K.T. Robbins, Department of Otolaryngology, Head and Neck Surgery, University of Tennessee, USA

In response to the increasing number of neck dissection procedures and growing list of names for these, an urgent need developed to standardize the terminology for neck dissection. Several classification systems have been offered over the past decade, both from European centers and the United States. In order to develop a classification system that would be widely accepted, the American Academy of Otolaryngology - Head and Neck Surgery through is Committee for Head and Neck Surgery and Oncology, developed such a system which was subsequently adopted by the American Society for Head and Neck Surgery (Robbins, K. T., Archives of Otolaryngolgy, 1991). Although this system was less intricate than some others that had been proposed, it was felt to serve the purpose of encouraging standarization, represent one that was logical and one that could be expanded based on future needs. The author will present this classification, outline its important aspects and comment on how this has been accepted within the United States. Concluding discussion will focus on current and subsequent usefulness of the system and what modifications may be useful for further improvement.

### 4.7
### The indication of supraomohyoidal cervical lymphnode exstirpation in the treatment protocol of oral cancer
B. Fleiner, A. Dunsche, Dept. of Oral and Maxillofacial Surgery, University of Kiel, Germany
Cervical lymphnode (CLN) metastases in oral squamous cell carcinoma depend on size, invasion and localization of the primary. According to the pretherapeutic clinical and sonographic findings the CLN extirpation can be planned as follows:
A. Tcis tumors of the oral cavity and T1 tumors of the lower lip are the only entities which do not always require CLN extirpation, if there is no evidence of CLN metastases (N0).
B. In T1 and T2 (N0) tumors of the anterior floor of the mouth a suprahyoidal extirpation including the jugulodigastric lymphnode is mandatory. Tumors of the posterior floor of the mouth require a supraomohyoidal lymphnode extirpation.
C. In all cases of T3 and T4 tumors or N1 to N3 staging disregarding the primary a complete ipsilateral neck dissection is necessary.
It has to be emphazised that unilateral tumors of the floor of the mouth and the tongue are liable to cause contralateral CLN metastates, due to which the treatment of the neck has to include the contralateral side. We perform a contralateral suprahyoidal or supraomohyoidal neck dissection in all cases which need a complete ipsilateral neck dissection even without evidence of contralateral CLN metastases. In order to evaluate this concept 105 ipsilateral complete neck dissections with contralateral suprahyoidal or supraomohyoidal CLN extirpations were analized. In five of these cases we found contralateral CLN metastases. This shows that even unilateral tumors of the floor of the mouth, which are treated with unilateral neck dissection according to their T- and N-staging should be treated with a contralateral suprahyoidal or supraomohyoidal CLN extirpation to eliminate contralateral CLN metastases.

### 4.8
### The value of contralateral supraomohyoid neck dissection
R. de Bree, C.R. Leemans, R. Tiwari, G.B. Snow, Dept. of ORL, University of Amsterdam, The Netherlands

**Introduction:** We have adopted a limited indication for supraomohyoid neck dissection (SOHND) in contralateral clinically N0 necks when the neck has to be entered for excision of a primary squamous cell carcinoma of the head and neck.
**Methods:** Between 1991-1996 we performed 44 SOHNDs with frozen-section examination of suspicious nodes. There were 40 lip/oral cavity and 4 oropharyngeal carcinomas. MRI, CT-scan and/ultrasound with cytology of enlarged lymph nodes were negative in all cases. Median follow-up was 24 months.
**Results:** Histopathological positive nodes were found in 4 of dissections (9.1%, all associated with ipsilateral positive nodes) and extranodal spread in one case. Radiotherapy was given to 81.8% of the necks. No neck recurrence occurred during follow-up.
**Conclusion:** Despite preoperative imaging and frozen-section analysis of nodes node-positivity in elective contralateral SOHNDs is 9.1%. These data indicate that our current policy of performing elective contralateral SOHND when the neck has to be entered for excision of the primary tumour in patients with tumours at or approaching the midline in the anterior oral cavity is safe.

### 4.9

### The role of selective neck dissection for node positive disease in patients with carcinoma of the upper aerodigestive tract

K.T. Robbins, Dept. of Otolaryngology, Head and Neck Surgery, University of Tennessee, USA

The indications of selective neck dissection as an operative procedure in the management of cervical metastases related to head and neck cancer is currently undefined. While there is minimal controversy with regard to its use in patients who have clinically negative nodal disease, its exact role for patients with positive nodal disease is far more controversial. Data will be presented to support the use of selective neck dissection in subsets of patients with clinical nodal disease. Data will also be presented to support the use of selective neck dissection as part of a multimodality regimen for patients with advanced nodal disease. The use of selective neck dissection continues to expand and we have yet to define its limitations, particularly as this relates to multimodality management of cervical metastases related to head and neck cancer.

### 4.11

### The outcome of a wait and see policy for the neck after negative ultrasound guided cytology results and follow-up with ultrasound guided cytology

M.W.M. van den Brekel, L.C. Reitsma, G.B. Snow, J.A. Castelijns, Dept. ORL, University of Amsterdam,The Netherlands

Ultrasound guided fine needle aspiration cytology has gained popularity in staging the clinically N0 neck in patients with head and neck carcinomas. On the one hand it can be used to upstage the N0 neck, thus enabling adequate treatment, on the other hand it can give more certainty that the neck is really free of metastases. The reported sensitivity of US-g-FNAC in the N0 neck ranges from 44% by Takes, 50% by Righi and 73% by our group. The specificity is 100% or slightly less as false positive cytology from squamous cell metastases in lymph nodes are very rare. A sensitivity of 73% in the N0 neck implicates that 73% of all palpably occult metastases can be detected. This means that if the initial risk of occult metastases is 40%, as in T2 tongue carcinomas, this risk deminishes to almost 10% after US-g-FNAC. As a consequence, the arguments to refrain from elective neck treatment gain strenght for certain patient populations. In our clinic, in the last 3 years we conducted a study in which US-g-FNAC negative patients, who in the past would have had an elective neck dissection, were now followed at regular intervals with palpation and US-g-FNAC. However, if the neck had to be entered for resection of the primary tumor, or for reconstruction of the defect, an elective neck dissection was carried out. In a series of 92 US-g-FNAC N0 patients, who were followed for 1-3 years 19 (21%) developed a neck node metastasis. Six of these 19 died of distant metastases or locoregional recurrence, one is alive with distant metastases and two died of unknown cause without metastases or locoregional recurrence. The characteristics of these patients will be discussed in terms of level, number and extranodal spread of metastases and delay of treatment. It can be concluded from these preliminary results that US-g-FNAC is false negative in a considerable number of patients and the reason for these false results as well as possible solutions will be discussed.

### 4.12

### Modified radical versus selective neck dissection in the clinically N0 neck

C.R. Leemans, G.B. Snow, Dept. Otolaryngology, Head & Neck Surgery, University of Amsterdam, The Netherlands

Surgical treatment of the clinically N0 neck remains controversial. The surgeon must decide whether a modified radical (MRND) or a selective neck dissection (SND) is justified. Both procedures preserve the integrity of the spinal accessory nerve but somewhat greater morbidity can be anticipated after MRND. The difference between the two procedures lies in the lymph node levels and structures that are left in-situ in SNDs. This dilemma between radicality of the procedure and sequelae deserves further analysis. Arguments against SND are possible spread directly to the lower deep cervical nodes (level IV) and the dependence on frozen-sections, and hence a skilled pathologist, to decide whether the procedure should be converted to a MRND. It has been well described that 25-40% of the electively dissected neck specimens contain only micrometastases, and that underassessment and missed pathological diagnosis do occur. Furthermore the procedure is less well standardised and possibly less complete in Level IIa and IV (if dissected). Radiotherapy is indicated in all pN+ cases after SND but this adjuvant therapy can be withheld in patients with minimal intranodal disease if a MRND has been performed. The argument supporting SND is its theoretical basis of predictable nodal metastatic spread for a given tumour. This concept seems to work statistically in the majority of patients, although recurrence rates in the dissected neck after SND seem to be somewhat higher than those reported for modified radical neck dissection. In our view there is a limited indication for SND on the clinically uninvolved contralateral side in patients with tumours at or approaching the midline, especially in those with anterior oral cavity carcinoma.

### 4.13

### Clinical/pathological assessment of neck metastases in glottic and supraglottic carcinomas

J. Czigner, L. Iván, M. Csanády, Dept. of Otorhinolaryngology, University of Szeged, Hungary

The management of squamous cell carcinoma of the supraglottic larynx requires a different philosophy than that of glottic cancer while the lymphatic system is involved at a much earlier phase and neck nodal metatases are much more common. The main question that remains is whether or not, in the patients in whom clinical metastases are not apparent, steps should be taken to eradicate occult metastases.

We undertook a retrospective review of 598 previously untreated consecutive patients from 1986 to 1996 with primary squamous cell carcinoma of the supraglottic or glottic larynx to ascertain the prevalence of neck node metastases and the rationale for elective and secondary neck dissections to reduce the number of END which may expose a large percentage of patients to unnecessary morbidity and expense.

Of the 598 patients 247 (41%) had neck dissection with operation of the larynx: 158 (60%) of the 265 patients with supraglottic tumor, 11 (5%) of the 225 patients with glottic cancer and 58 (56%) of the 104 patients with transglottic cancer.

69 (54%) of the primary 128 neck-dissection proved to be histologically positive in the supraglottic group, 5 (29%) of the 17 primary ND were metastatic histologically in the group of glottic carcinoma and 32 (64%) of the 50 ND were found having carcinoma metastases in the lymph nodes in the group of transglottic tumors. As regarding the secondary radical neck dissections 30 (sRND) (36%) were performed of the 83 previously non-operated neck because of developed metastases, all proved to be positive histologically in the supraglottic group. 19 (9%) of the 208 non-dissectioned neck required SRND in the glottic group and 16 (30%) of the 54 earlier negative neck in the transglottic group necessitated SRND.

In our study the more precise preoperative assessment (CT, MRI, Sonography) increased the number of the primary neck dissections in the cases of clinically N0 necks and also revealed a relative high, negative histological finding of lymph nodes (clinically false positive cases).

The increased number of elective neck dissections (END) did not reduce the frequency of the development of secondary metastases in primary N0 cases.

Our study did not establish the value of the increased number of elective neck dissections in the clinically N0 cases, especially in the group of glottic cancer. All the teams of head and neck surgeons performing elective neck dissection must reassess their rationale or restudy their occult disease rate with computed tomography, magnetic resonance imaging and sonography for more precise selection for END.

4.14

**Neck dissection for laryngeal carcinoma**

K. Yoshino, T. Sato, T. Fijii, K. Inakami, S. Nishimoto, M. Nagahara, J. Okita, C. Momohara, Dept. of Otolaryngology, Head and Neck Surgery, Osaka Medical Center for Cancer and Cardiovascular Diseases, Japan

A retrospective analysis of previously untreated 1,045 patients with squamous cell carcinoma of the larynx, curatively treated at our clinic from 1979 to 1993, was performed. The goals of this study were to clarify the optimal neck dissection (ND) procedure and its indication for laryngeal carcinoma. The rates of occult neck metastasis according to site, laterality and T-stage > were examined to determine the indication of elective ND for the clinical N0 > neck. We considered that if the probability of occult neck metastasis was greater than 20% , elective ND was warranted. The results suggested that the indication for elective ND was as follows: supraglottis / lateral(L)-type T3, 4, median(M)-type T2, 3, 4, transglottis / L-type T2, 3, 4, M-type T3, 4, and glottis / L-type T4, M-type T3, 4; bilateral ND were necessary in all except transglottis / L-type. Our procedures for ND were classified into the following five types; standard radical ND(RND), modified RND(MRND), lateral ND(LND), lymphadenectomy(LAD) and paratracheal ND(PND). The recurrence rates in the N0 neck treated by LND were 4/107(4%) for the ipsilateral side of L-type, 1/23(4%) for the contralateral side and 3/43(7%) for M-type; those in the N+ neck were 3/55(5%) for N1, 0/5(0%) for N2a and 2/14(14%) for N2b (the N2c neck was reclassified on each side, e.g., N1 on the right and N2a on the left). By the comparison of the recurrence rates and morbidity between ND procedures, the following procedures were considered as optimal: LND for N0, N1, smaller N2a, MRND or RND for larger N2a, N2b and RND for N3. A relationship was examined between subglottic extension of carcinoma and metastasis in the paratracheal region; the former was divided into five grades. The rates of metastasis were greater than 20% in grade 3, 4, in which PND was indicated. Our technique of PND was suggested to be appropriate.

4.15

**Surgical treatment of regional metastasis of larynx cancer**

D.F. Shamsiev, Dept. of Otorhinolaryngology, Head and Neck Surgery, Tashkent Medical Institute, Uzbekistan

Crile's operation is associated with different delayed postoperative complications, such as neck deformation, muscular atrophia, persistent shoulder pain syndrome, neurinoma formation. This investigation was performed in order to improve postoperative period and to prevent some complications.

Histological investigation of 24 micropreparations, including neck lymph nodes with fatty tissue and fascia, clavisternomastoid muscle, „internal" jugular and submandibular salivary gland did not show infiltration of malignant tumor into clavisternomastoid muscle.

Taking into account this fact we performed operations in cases of regional metastasis of larynx cancer with preservation of clavisternomastoid muscle.

Clinical investigation of 46 patients who underwent Crile's operation with preservation of clavisternomastoid muscle revealed that this operative intervention had the following advantageous features.

1. Absence of cosmetic neck defects.
2. Preservation of clavisternomastoid muscle is a good defense for uncovered neurovascular bundle.
3. Postoperative pain syndrome is less marked.

In this connection preservation of clavisternomastoid muscle as well as maximum spare of accesory nerve allow to obtain better results of postoperative period and to prevent a number of complications.

4.16

**Laryngo-pharyngeal carcinoma and state of metastasis - analysis of 1000 cases (1985-1995 at Dortmund municipal hospital)**

F. Jahnke, K. Koeser, G. Bertram, Dept. of Otorhinolaryngology, Municipal Hospital Dortmund, Germany

Base of an adequate therapy of carcinoma of the upper aerodigestive tract are exact knowledge of local cancer spread, lymphnode status and the exclusion or proof of distant metastasis. Technical examinations for detection of distant metastases are chest-x-ray, bone scan and abdominal ultrasound.

Data about the frequency of distant metastasis at the time of primary diagnosis are few.

The aim of this study was to find valuable rates about metastatic spread and to define criteria for the use of these technical examinations.

The medical records of 1000 patients with squamous cell carcinoma of larynx and hypopharynx of our hospital examined first time during a 10 year period from 1985 to 1995 were analized.

The highest rate of metastasis was found in chest-x-ray. No patient of undergoing abdominal ultrasound was foudcnd to have hepatic metastases. In bone scanning only one patient was found to have metastatic disease.

4.17

**The distribution of lymph node metastases in supraglottic squamous cell carcinoma: therapeutic implications**

P. Nicolai, L.O. Redaelli de Zinis, D. Ghizzardi, D. Tomenzoli, N. Nassif, A.R. Antonelli, Dept. of Otolaryngology, University of Brescia, Italy

A series of 402 consecutive patients treated between 1983 and 1995 for supraglottic squamous cell carcinoma was analyzed with regard to the distribution of lymph node metastases. Neck dissection had been performed concomitantly to surgery on the primary lesion, which consisted of supraglottic laryngectomy in 179 patients, supracricoid laryngectomy in 13, and total laryngectomy in 210. In 199 patients, the neoplasm did not extend beyond the midline and was therefore defined as lateralized. The distribution according to pT category was: 10 T1, 103 T2, 184 T3, 105 T4. The overall incidence of lymph node metastases was 40%, and the frequency proportionally increased with T category from 10% in T1 to 57% in T4 ($p < 0.001$). There were occult metastases in 26% of patients, with a distribution in relation to T category ranging from 20% in T2 to 40% in T4 ($P = 0.02$). The incidence of bilateral metastases was statistically higher in central than in lateralized lesions (20% vs. 5%; $p < 0.001$). Occult metastases in the contralateral side of the neck were detected in 3% of lateralized neoplasms. The distribution of metastases according to lymph node levels was as follows: 0.5% in level I, 82% in level II, 41% in level III, and 12% in level IV. Level V nodes were never involved, whereas metastatic lymph nodes were present in level I and IV only in conjunction with level II or III involvement. Elective treatment of the neck is currently performed at our institution only when the probability of occult metastases exceeds 15%. Based on the data herein presented, elective neck dissection is not indicated in T1 supraglottic lesions or in the contralateral side of the neck for lateralized lesions. Since no occult metastases were detected in level I or V, the management of choice for the clinically negative neck is a selective dissection limited to levels II-IV. We still prefer to include level V in the dissection whenever there is clinical, radiological or intraoperative evidence of lymph node metastases at any other level.

### 4.18

**Lymph node metastases from laryngeal and pharyngeal carcinomas. Calculation of burden of metastasis and its impact on prognosis**

J. Jakobsen[1], O. Hansen[2], K.F. Jørgensen[1], L. Bastholt[2], [1]Dept. of ORL, [2]Dept. of Oncology, University of Odense, Denmark

During the period 1965-92, a total of 1069 patients, 739 patients with newly diagnosed laryngeal and 330 patients with pharyngeal cancer, were seen at the Centre for Head and Neck Cancer, Odense University Hospital. One percent (5/499) of the glottic cases and 29 percent (68/232) of the supraglottic cases had primary lymph node metastases. When the primary tumour was located endolaryngeally, the frequency of metastases was highest at the inlet of the larynx - 38 percent - decreasing gradually in distal direction to 1 percent at the level of the vocal cords. The frequency of metastases among patients with pharyngeal carcinomas was 66 percent (218/330). All patients had primary irradiation, apart from 10 patients having primary surgery. Calculation of the burden of lymph node metastases was based on the volume formula of an ellipse in 280 out of 291 patients with metastases. The calculated volumes ranged from 1 to 1413 $cm^3$ and were divided into 3 groups according to size. A Cox multivariat regression analysis using crude and disease specific survival as endpoint revealed the burden of metastasis to be an independent, prognostic factors.

### 4.19

**Markers for assessment of the neck in laryngeal carcinoma**

R.P. Takes[1], R.J. Baatenburg de Jong, E. Schuuring[2], J. Hermans[3], S.V. Litvinov[2], J.H.J.M. van Krieken[2], [1]Dept. of Otolaryngology, [2]Dept. of Pathology, [3]Dept. of Biostatistics, University of Leiden, The Netherlands

**Introduction:** In recent years, imaging techniques for assessment of regional metastasis in patients with head and neck squamous cell carcinoma have improved. However, it is still impossible to detect small metastastic deposits. In this study it is investigated whether it is possible to assess regional metastasis in patients with laryngeal carcinoma by studying features of the primary tumor.
**Materials and methods:** Several histological features and biological markers were examined in 31 laryngeal carcinomas. The markers (PCNA, p53, Rb, MYC, BCL-2, EGF, EGFR, neu, nm23, desmoplakin, N-CAM, Ep-CAM, E-cadherin, CCND1 and EMS1) were selected on their putative role in the process of metastasis and were studied using immunohistochemical and/or Southern blot techniques.
**Results:** Inflammatory reaction surrounding the tumor, eosinophilic infiltration, immunostaining for Rb and for Ep-CAM and amplification of CCND1 and EMS1 correlated with nodal metastasis. The combination of some of these markers resulted in a superior accuracy in assessing nodal metastasis.
**Conclusions:** These results indicate that it is possible to predict and exclude regional metastasis by studying features of the primary tumor. When these results are confirmed in a larger series, biological markers may be powerful diagnostic tools with great impact on clinical decision making.

### 4.20

**Simultaneous bilateral Crile operation in patients with laryngeal cancer**

Z. Szmeja, A. Kruk-Zagajewska, Dept. of Otolaryngology, University of Poznan, Poland

Complications following a simultaneous bilateral Crile operation are caused by the soft tissue structures removal in the neck, especially in the venous area, parts of lymphatic and nervous system.
The aim of the paper was the evaluation of nine patients condition in whom total laryngectomy was performed together with simultaneous, bilateral Crile operation. Their age ranged between 42-60 years. All of them were in the 4th degree (T4N3M0) of laryngeal cancer clinical advancement. After the operation attention was paid to edema and cyanosis of the face, blood pressure, brain edema, dimness of vision and pain on swelling of the shoulder.
Simultaneous bilateral Crile operation in justified oncological cases (N3) is possible to carry out. Slow blockade of internal jugular veins by metastatic lymph nodes and neoplastic cell embolia cause the developement of collateral venous circulation and providing the adaptation of brain tissue. A close cooperation with anesthesiologists, neurosurgeons and ophthalmologists is essential in the post operative treatment.
All our patients underwent the simultaneous bilateral Crile operation without any complications.

### 4.21

**Management of parastomal neoplasm of laryngeal carcinoma**

F.U. Metternich, T. Brusis, Dept. of Otorhinolaryngology, Head and Neck Surgery, Clinic Holweide, Cologne, Germany

**Background:** Parastomal neoplasm after total laryngectomy for laryngeal carcinoma represents one of the most formidable therapeutic problems encountered by the head and neck surgeon. Studies about the management of parastomal neoplasm have been controversial. Conservative management concepts are radiotherapy or chemotherapy. Surgery includes a local tumor exstirpation in combination with a radiation therapy or an extensive tumorresection with a flap reconstruction. **Patients:** To clarify the controversial aspects of parastomal neoplasm management, a systematic management analysis was performed using data from 10 patients who developed parastomal neoplasm. **Results:** Parastomal neoplasm development does not correlate neither with the initial operative technique of laryngectomy nor with a postoperative radiotherapy or chemotherapy. In comparison to a moderate parastomal neoplasm surgery or a conservative management an extensive neoplasm resection does not show a significant influence on the patient's survival time especially in cases of an advanced tumor growth. The long-term prognosis remains poor with an average survival time of 7.8 months. **Conclusion:** In cases of an advanced parastomal tumor growth an extensive surgical resection does not improve the patient's long-term prognosis. An amelioration of the prognosis could be obtained by an early detection of parastomal tumor growth. The problem of parastomal neoplasm can only be solved by clearing up the neoplasm etiology.

### 4.22
### Progress in voice restoration after treatment of larynx-hypopharynx cancers and their metastases

G. Lichtenberger, Szent Rókus Hospital, Budapest, Hungary

The Blom-singer puncture and implantation of voice prosthesis were without doubt pioneer methods. These treatments were initially performed after simple laryngectomy. The techniques have been later used by patients suffering from enlarged larynx-hypopharynx cancers and their metastases as well. It became sure, that the voice prosthesis implantation has its place after neck dissection combined with laryngo-pharyngectomy, too. The voice prosthesis implantation might also be successful after reconstruction of pharyngeal defects with PMM-flap, gastric pull up technique and free jejunum graft transplantation. To predict the results, the insufflation and Lidocain tests proved to be advantageous. The number of successfully implanted patients could be increased by using the cricopharyngeal myotomy. The application of new and improved types of voice prostheses ensured favourable results. However there was not a real progress in the puncture technique. After the Blom-Singer puncture occured in some cases small or even severe complications, too. This technique proved especially to be very difficult, when there were scars behind the stoma. This scar formation is much more frequent after the operation of enlarged larynx-hypopharynx cancers and their metastases, due to prolonged and secondary wound healing, than after simple laryngectomy.
These problems urged the author to develop a simple endo-extra-oesophageal puncture technique instead of the Blom-Singer method. He performs his simplified puncture technique by forming the fistula with the help of his endo-extralaryngeal needle carrier and a pointed metal cone with a needle built in fixed to the catheter.
The operation is performed under JET anaesthesia that makes the patient free from any pains and leaves enough room for the surgeon in the area of the stoma.
The pharynx is opened with the laryngoscope that is then led up to the entrance of the oesophagus. Through the laryngoscope the distal end of the endo-extralaryngeal needle carrier is led into the oesophagus. The instrument is pushed forward as long as its distal bent end is palpable in the upper third of the tracheostoma. The needle with the thread (Prolen 2/0) is pushed through from inside out in the upper third of the tracheostoma. A double wire forming a loop is led through the pointed metal cone (in which there is a needle built in) and the catheter, and it is tied behind a conterfixing pierced ball. The Prolen 2/0 leading thread is then knotted with the wire. By pulling the thread and the wire the pointed end of the metal cone with the needle built in, perforates the soft parts and pulls the catheter with itself. After the fistula has been prepared for the implantation of the prosthesis, the metal cone, the thread and the wire are cut off the end of the catheter.
The advantage of this endo-extra-oesophago-tracheal puncture technique is, that the risk of injuring the back-wall of the oesphagus or pharynx is eliminated. After this procedure the voice prosthesis can be placed easily in the fistula on conventional way.

### 4.23
### Management of the neck in patients with salivary gland malignancies

C.G. Mahnke, H. Rudert, B.M. Lippert, J.A. Werner, Dept. of Otorhinolaryngology, Head and Neck Surgery, University of Kiel, Germany

Malignancies of the salivary glands constitute around 7% of all malignant epithelial tumors of the head and neck. In these tumors the histology varies greatly as does the rate of metastases to the cervical lymph nodes. The purpose of this presentation is to analyse literature data and clinical data of patients treated at the University of Kiel to more precisely define the rate of locoregional metastasis and to establish guidelines for the indication of neck dissection.
The charts of all patients treated for malignancies of the salivary glands at the Dept. of Otorhinolaryngology, Head and Neck Surgery, University of Kiel, Germany, between 1954 and 1993 were analysed retrospectively with regard to the occurrence of cervical lymph node metastasis. The literature data on salivary gland malignancies was investigated.
The rate of metastasis depended on the histologic type of tumor. It is highest in mucoepidermoid carcinomas, oncocytotic carcinomas, salivary duct carcinomas and undifferentiated carcinomas. In these tumors lymphatic spread is found in up to 80%. Tumors with a rate of metastasis around 10% are adenoidcystic carcinomas, polymorphic low-grade adenocarcinoma, basalcell-adenocarcinoma and myoepithelial carcinoma. Given the high variation in the rate of nodal spread of salivary gland malignancies the histologic typing of the primary tumor has to be taken into consideration, particularly when discussing the need for neck dissection in the N0 neck. In patients with malignancies of the parotid gland elective neck dissection should accordingly be performed in cases of squamous cell carcinoma, mucoepidermoid carcinoma (of low and intermediate differentiation), adenocarcinoma, salivary duct carcinoma and carcinoma in a pleomorphic adenoma. Also, since the first lymphatic drainage is located intraglandular or intraregional one has to include these lymphatics in the surgical approach.

### 4.24
### Management of the neck in patients with skin cancer

R.S. Weber, G. Tucker, Dept. of Otorhinolaryngology, Head and Neck Surgery, University of Pennsylvania, USA

Basal and squamous cell carcinoma of the skin of the head and neck is most frequently a local management problem. Nevertheless regional lymph node metastases primarily from squamous cell carcinoma of the skin do occur in some patients. Multiple factors related to the primary tumor may predict patients at greater risk for regional spread. These include primary site, recurrence after initial treatment, lesions with deep invasion into underlying tissues and poorly differentiated histology. Among patients with identifiable risk factors, regional lymph node assessment through physical examination and radiologic imaging is important to properly stage the disease and plan surgical resection. Lymph node basins at risk are predictable based on the site of the primary; however, the parotid and upper cervical lymph nodes are of primary importance. This presentation will review the indications and surgical management of regional lymph nodes including parotidectomy and selective versus comprehensive neck dissection for the management of regional metastases. In addition the role of radiation therapy as a surgical adjunct will be addressed.

### 4.25
### Management of cervical lymph nodes in thyroid cancer

H. Goepfert, Dept. of Head and Neck Surgery, MD Anderson Cancer Center, Houston, USA

There is significant controversy as to the most appropriate therapy to the regional lymph nodes in differentiated and medullary thyroid cancers.

The different sentinel and secondary nodal groups have been defined and a consistent, rational plan of surgical management should be applied.

Based on a review of pertinent literature and the experience accrued at UTMDACC a diagnostic and treatment plan will be presented. For differentiated carcinoma a central compartment (pre- and paratracheal up to the jugular vein) dissection should be done and more extensive nodal dissections should be tailored to the findings at the time of surgery and the additional risk factors of the individual patient. A more aggressive surgical attitude (modified neck dissection) is recommended for patients with medullary carcinoma, both in the elective as well as the therapeutic settings.

The potential value of adjuvant therapies (radioactive iodine 1-131 and/or external beam radiotherapy) as well as the principles of short and long-term follow-up will be discussed.

### 4.27

### Management of carcinoma of the supraglottic larynx: evolution, current concepts, and future trends

E.N. Myers, A. Alvi, Dept. of Otolaryngology, University of Pittsburgh, USA

The treatment of cancer of the supraglottic larynx has undergone an evolution. Better understanding of the anatomy and biology of cancer in this anatomic site has enabled surgeons to devise effective oncologic strategies while making every effort to preserve the function of the larynx. Certain recent concepts and changing trends have emerged in the treatment of cancer of the supraglottic larynx, including the treatment of the neck, significance of extracapsular spread of tumor in cervical lymph nodes, and conservation laser surgery. Snyderman et al. reported the prognostic significance of extracapsular spread in patients with cancer of the supraglottic larynx. Lutz et al. reported the results of our experience with the treatment of 202 patients. The review verified the significant risk of bilateral neck disease in these patients even with adjuvant radiation therapy. Accordingly, since 1990, all patients having cancer of the supraglottic larynx in the Department of Otolaryngology at the Universtiy of Pittsburgh have been treated with bilateral neck dissections. The use of adjuvant radiation therapy has been based on the presence of extracapsular spread. This study documents the oncologic effectiveness of this treatment and confirms the efficacy of bilateral neck dissections in an attempt to control neck disease and the prognostic significance of extracapsular spread. We review the evolution of the treatment of cancer of the supraglottic larynx, present our results, and consider innovative surgical approaches.

### 4.28

### Paratracheal lymph node metastasis in carcinoma of the laryngopharynx and cervical esophagus

R.S. Weber, J. Marvel, P. Smith, P. Hankins, P. Wolf, H. Goepfert, Philadelphia, Houston, USA

Paratracheal lymph node metastasis (PTLN) has been implicated as an etiologic factor in peristomal recurrence following laryngectomy. To determine the incidence of paratracheal lymph node metastasis and the prognostic significance of this finding the records of 645 patients treated at the University of Texas M.D. Anderson Cancer Center who underwent total laryngectomy between 1978 and 1990 for squamous cell carcinoma of the larynx, hypopharynx and cervical esophagus were reviewed. Criteria for inclusion in the study were 1) surgical description of PTLN dissection, 2) hemi- or total thyroidectomy and 3) pathologic identification of paratracheal lymph nodes. One hundred forty one patients met this requirement and comprised the cohort analyzed. Primary site of origin was larynx 64.5%, hypopharynx 25.5%, and cervical esophagus 9.9%. Subglottic extension was identified in 33% of patients. The mean number of PTLNs removed was 3.9 (range 1-30), and metastasis was present in 29 patients (20.5%). Factors associated with paratracheal lymph node metastasis were site of origin and subglottic extension. This paper will present the technique of paratreacheal lymph node metastasis, factors associated with spread to this nodal basin and the impact on peristomal recurrence.

### 4.29

### New perspective on controversies in the management of the N0-neck

S.L. Collins, K. Muzaffer, Dept. of Otolaryngology, Head and Neck Surgery, Loyola University Chicago, USA

Three major factors influence treatment outcome for the $N_0$ neck; initial cancer control, salvageability of recurrent disease, and quality of life (QL). Only the first is typically considered in the perennial controversy over optimal treatment of occult cancer in the head and neck (HNC) literature. This presentations assesses failure rates, salvageability of recurrent disease, and survival (overall and cancer-free) related to treatment modality (elective neck dissection [END], elective neck irradiation [ENI] or observation [OBS]) in a group of 425 patients with HNSCC and $N_0$ necks with a median follow-up of 33-48 months in whom the primary tumor remained controlled. Additional END patients also received partial neck RT in the course of treating the primary tumor with combination therapy.

**Results:**

| | Observation | END | ENI | END+RT |
|---|---|---|---|---|
| Failure rate | 7%(17/244) | 9.5%(2/21) | 18%(17/96) | 14%(9/64) |
| Salvage attempted | 16/17 | 2/2 | 13/17 | 4/9 |
| Salvage successful | 63%(10/16) | 50%(1/2) | 15%(2/13) | 25%(1/4) |
| Cancer free survival | 75% | 71% | 53% | 62% |

Recurrent cancer cannot be reliably salvaged in previously irradiated necks. Patients receiving ENI failed twice as often as END patients and were less than ⅓ as salvageable. The initial control, salvageability, and survival rates were best in the OBS group. This data, in combination with QL considerations tips the scale in favor of OBS or END for the $N_0$ neck, instead of ENI. Selection criteria for END(s) vs. OBS are presented. Additional factors against ENI include potential selection of aggressive treatment-resistant tumor cell variants, radiation-related promotion of cancer growth in adjacent normal tissue and accelerated arterial wall thickening which may increase the likelihood of stroke in this predisposed population.